# Tegafur-Uracil versus 5-Fluorouracil in Combination with Cisplatin and Cetuximab in Elderly Patients with Recurrent or Metastatic Head and Neck Squamous Cell Carcinoma: A Propensity Score Matching Analysis

**DOI:** 10.3390/biology10101011

**Published:** 2021-10-08

**Authors:** Meng-Che Hsieh, Chih-Chun Wang, Chuan-Chien Yang, Ching-Feng Lien, Chien-Chung Wang, Yu-Chen Shih, Shyh-An Yeh, Tzer-Zen Hwang

**Affiliations:** 1 Department of Hematology-Oncology, E-Da Cancer Hospital, Kaohsiung City 82445, Taiwan; ed111216@edah.org.tw; 2 College of Medicine, I-Shou University, Kaohsiung City 82445, Taiwan; ed103747@edah.org.tw (C.-C.W.); ed101194@edah.org.tw (C.-C.Y.); ed106789@edah.org.tw (C.-F.L.); ed108936@edah.org.tw (C.-C.W.); ed109324@edah.org.tw (Y.-C.S.); ed101362@edah.org.tw (S.-A.Y.); 3 Department of Otolaryngology, E-Da Hospital, Kaohsiung City 82445, Taiwan; 4 Department of Otolaryngology, E-Da Cancer Hospital, Kaohsiung City 82445, Taiwan; 5 Department of Radiation Oncology, E-Da Hospital, Kaohsiung City 82445, Taiwan

**Keywords:** elderly patients, recurrent or metastatic head and neck squamous cell carcinoma, tegafur–uracil, cetuximab, survival

## Abstract

**Simple Summary:**

Elderly patients with recurrent or metastatic head and neck squamous cell carcinoma are a unique subset because they are at increased risks of miserable prognosis. Although cisplatin, 5-fluorouracil plus cetuximab (EXTREME) is the most commonly used regimen, chemotherapy de-escalation strategy was suggested in elderly patients due to toxicity. Herein, an oral tegafur–uracil is usually substituted for 5-fluorouracil and combined with cisplatin plus cetuximab (UPEx) as a novel agent for elderly patients with recurrent or metastatic head and neck squamous cell carcinoma. The median progression-free survival was 5.4 months in UPEx and 5.8 months in EXTREME (*p* = 0.451). The median overall survival was 10.8 months in UPEx and 10.2 months in EXTREME (*p* = 0.807). Grade 3/4 adverse events were much fewer in UPEx than in EXTREME (*p* < 0.001). Our study demonstrated that UPEx is effective with improving safety profiles. We suggested UPEx might be a better treatment option for elderly patients with recurrent or metastatic head and neck squamous cell carcinoma.

**Abstract:**

There are increasing incidences of elderly patients with recurrent or metastatic head and neck squamous cell carcinoma (R/M HNSCC). However, the treatment is not yet established. We conducted a propensity score matching analysis to evaluate the efficacy and safety of tegafur–uracil versus 5-fluorouracil in combination with cisplatin plus cetuximab in elderly patients with R/M HNSCC. Elderly patients with R/M HNSCC treated with cetuximab-containing chemotherapy were recruited into this study. In order to reduce the selection bias, propensity score matching was performed. Kaplan–Meier curves were plotted for progression-free survival (PFS) and overall survival (OS). Toxicities were graded according to the National Cancer Institute’s Common Terminology Criteria V3.0. After propensity sore matching, 54 patients with tegafur–uracil, cisplatin plus cetuximab (UPEx), and 54 patients with 5-fluorouracil, cisplatin plus cetuximab (EXTREME) were identified. The median PFS was 5.4 months in UPEx and 5.8 months in EXTREME (*p* = 0.451). The median OS was 10.8 months in UPEx and 10.2 months in EXTREME (*p* = 0.807). The overall response rate (ORR) and disease control rate (DCR) were insignificant in both arms, accounting for 61% versus 59% (*p* = 0.680) and 72% versus 70% (*p* = 0.732) in the UPEx arm and the EXTREME arm, respectively. A multivariate analysis showed that age and ECOG PS were, independently, predictors. Grade 3/4 adverse events were much fewer in UPEx than in EXTREME (*p* < 0.001). Both cetuximab-containing chemotherapies are effective in elderly patients with R/M HNSCC. Safety profiles are improved when tegafur–uracil is substituted for 5-fluorouracil. Further prospective studies are warranted to validate our conclusions.

## 1. Introduction

Head and neck squamous cell carcinoma is the sixth most common cancer worldwide [1]. The Global Burden of Disease study estimated 890,000 new cases in 2017, representing 5.3% of all cancer patients [2]. The prognosis of patients with recurrent or metastatic HNSCC (R/M HNSCC) remains poor, with median overall survival (OS) at around 12–14 months [3]. Elderly patients with R/M HNSCC are a unique subset because they are at increased risk of poor prognosis. Statistically, half of patients were newly diagnosed to have head and neck cancer at ages over 60 years, and 70% of these patients died at ages over 70 years [4]. Management can be challenging in this group due to the presence of comorbidities, disabilities, polypharmacy, geriatric symptoms, and social issues. Therefore, the treatment of elderly patients with R/M HNSCC deserves close attention and multi-disciplinary consensus.

Current international guidelines suggest cetuximab-containing regimen is the standard chemotherapy for patients with R/M HNSCC [5]. The pivotal EXTREME study demonstrated that cisplatin, 5-fluorouracil (5-FU) plus cetuximab, followed by cetuximab maintenance weekly improved survival significantly, as compared with cisplatin plus 5-FU [6]. The overall response rate (ORR) increased by 16%, median progression-free survival (PFS) was prolonged by 2.3 months, and median OS extended 2.7 months after this combination treatment. However, the safety profiles reported 82% grade 3 or 4 adverse effects, which might be a major issue for elderly patients.

To date, there are no standard treatments for elderly patients with R/M HNSCC. Given that the incidences of elderly patients with R/M HNSCC are increasing, there are urgent unmet needs. Although EXTREME is the most commonly used regimen, chemotherapy de-escalation strategy was suggested in some elderly patients due to toxicity. At our institute, oral tegafur-uracil (UFUR; TTY Biopharm, Taiwan) is usually substituted for 5-FU and combined with cisplatin plus cetuximab (UPEx). UFUR is composed of tegafur and uracil in a 1:4 molar ratio. Tegafur is an orally bioavailable prodrug of 5-FU, and uracil is an orally administered fluoropyrimidine inhibitor of dihydropyrimidine dehydrogenase, which can increase the effect of 5-FU with a low toxicity profile [7]. Herein, we conducted a propensity score matching analysis to investigate the oncologic outcomes of cetuximab-containing chemotherapy in elderly patients with R/M HNSCC.

## 2. Materials and Methods

### 2.1. Patients

Patients who were diagnosed to have R/M HNSCC from 2017 to 2020 at E-Da Hospital were reviewed. Elderly patients with R/M HNSCC, treated with UPEx or EXTREME as first-line chemotherapy, were enrolled into our study. The patients’ clinical and laboratory data were retrieved from medical records. Inclusion criteria were elderly patients with age over 70 years, pathologically confirmed R/M HNSCC, including cancers of the oral cavity, oropharynx, hypopharynx, and larynx with a metastatic or recurrent disease. Exclusion criteria were previous history of cetuximab or UFUR before R/M HNSCC, not first-line cetuximab-containing regimen, rapid progression within six months after curative platinum based concurrent chemoradiotherapy, and irregular follow-up intervals. This was a retrospective study, which was exempt from requiring consent. This study was approved by the E-Da Hospital Institutional Review Board (EMPR-109-089) and was conducted in accordance with the Declaration of Helsinki.

### 2.2. Treatment Methods

For UPEx treatment, patients were treated with a 2-week cycle of oral tegafur 200 mg twice per day for 14 days of each cycle, oral leucovorin 100 mg twice per day for 14 days of each cycle, cisplatin 35–50 mg/m^2^ on day 1 of each cycle plus cetuximab 400 mg/m^2^ loading at day 1 of cycle 1 and then 250 mg/m^2^ weekly on subsequent administration. For EXTREME treatment, patients were treated with a 4-week cycle of cisplatin 70–100 mg/m^2^ on day 1 of each cycle and 5-FU 700–1000 mg/m^2^ on day 1–4 of each cycle plus cetuximab 400 mg/m^2^ loading at day 1 of cycle 1 and then 250 mg/m^2^ weekly on subsequent administration. Dose modification could be adjusted according to patients’ comorbidities and treatment adverse effects. The prophylactic medication consisted of an anti-anaphylactic, anti-emetic, and hydration. Carboplatin was used subsequently in substitution for cisplatin if renal toxicity developed. Computed tomography was arranged for evaluation about the treatment response every 3–4 months. Treatment was continued in responding or stable patients until disease progression or unacceptable toxicity.

### 2.3. Statistical Analysis

Basic characteristics were retrieved from a medical chart review and presented with frequencies. The differences between groups were compared with chi-square test. Statistical analyses were performed using SPSS. In order to reduce the selection bias, propensity score approach was performed using logistic regression models via nearest neighbor approach with a caliper of 0.2. Matching was performed without replacement. Balanced covariates included gender, age, Eastern Cooperative Oncology Group Performance Status (ECOG PS), smoking, primary tumor location, previous treatment before recurrent/metastasis, and disease status. The oncologic outcomes were summarized with progression-free survival (PFS), overall survival (OS), overall response rate (ORR), and disease control rate (DCR). Progression-free survival (PFS) was measured from the first day of chemotherapy administration until the date of tumor progression or final follow-up, while overall survival (OS) was calculated as the time from the first day of chemotherapy administration until the date of death from any cause or final follow-up. Objective response criteria in the tumors were also evaluated according to the RECIST 1.1 guidelines, including complete response (CR), partial response (PR), stable disease (SD), and progressive disease (PD), ORR was defined as CR plus PR, and DCR was defined by CR, PR, plus SD. Kaplan–Meier curves were depicted for survival. We also conducted Cox regression multivariate analysis using “enter” selection to adjust the effects of potential confounders. All *p* values were two sided and considered to have significance if *p* values < 0.05. Toxicities were graded according to the National Cancer Institute’s Common Terminology Criteria V3.0.

## 3. Results

### 3.1. Patient Characteristics

A total of 152 patients were enrolled into our study for oncologic outcomes evaluation. The median age of our patients was 72 years. Baseline characteristics were presented in Table 1. In general, 23% of patients were older than 72 years, and 47% patients had an Eastern Cooperative Oncology Group Performance Status (ECOG PS) of 2. Most patients were male (93%). More than 90% of our patients were current smokers or former smokers. The majority of primary tumor location was hypopharynx (39%), followed by oral cavity (37%), oropharynx (20%), and larynx (4%). Nearly 60% of our patients received radical surgery, and 87.5% of our patients underwent chemoradiotherapy before their recurrent or metastatic disease. After recurrence or metastasis, 80% of patients had distant metastasis with or without local recurrence, while the remaining had local recurrent disease only. After propensity sore matching, 54 patients with UFUR and platinum plus cetuximab (UPEx) and 54 patients with 5-fluorouracil and platinum plus cetuximab (EXTREME) were identified. All basic characteristics including gender, age, ECOG PS, smoking status, primary tumor location, previous history of curative treatment, and disease status were well balanced between the two treatment arms.

### 3.2. Survival Outcomes

**Figure 1:**
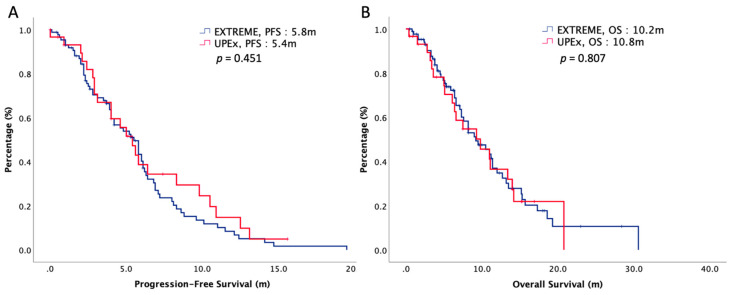
(**A**) Progression-free survival and (**B**) overall survival of 108 elderly patients with R/M HNSCC, stratified by chemotherapy regimen.

The median follow-up interval was 9.7 months. At the end of our study, 75% of our patients died, and cancer (96%) was the main cause of death. The oncologic outcomes were summarized in Table 2. The median PFS was 5.4 months in UPEx and 5.8 months in EXTREME (*p* = 0.451). The median OS was 10.8 months in UPEx and 10.2 months in EXTREME (*p* = 0.807). The survival curves of PFS and OS are plotted in Figure 1. The ORR and DCR were both insignificant in both arms, accounting for 61% versus 59% (*p* = 0.680) and 72% versus 70% (*p* = 0.732) in the UPEx arm and the EXTREME arm, respectively. Cox regression multivariate analyses with PFS and OS for prognostications were depicted in Table 3. Multivariate regression analysis demonstrated that age > 72 and ECOG PS 2 were independently negative predictors that correlated with survival. The chemotherapy regimens, UPEx or EXTREME, did not have a significant impact on survival.

**Figure 2:**
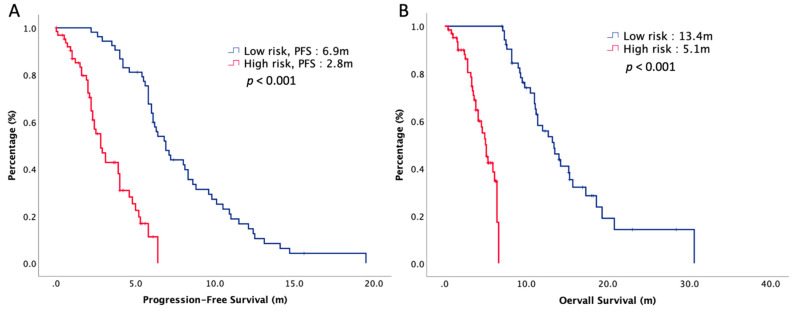
(**A**) Progression-free survival and (**B**) overall survival of 108 elderly patients with R/M HNSCC, stratified by risk groups.

Based on these two significantly prognostic factors, age and ECOG PS, we stratified our patients into a low-risk group and a high-risk group. The low-risk group indicated patients with 0–1 prognostic variable, while the high-risk group indicated those with two variables. In total, 53 patients were classified as low-risk group, and 55 patients were classified as high-risk group. PFS and OS of low-risk and high-risk groups are presented in Figure 2. The median PFS and OS were 6.9 months versus 2.8 months (*p* < 0.001) and 13.4 months versus 5.1 months (*p* < 0.001) in the low-risk group and the high-risk group, respectively.

### 3.3. Safety Profiles

The treatment-related adverse events (AEs) were significantly different between two treatment arms. AEs ≥ grade 3 were reported in Table 4. Overall, grade 3/4 AEs are much fewer in UPEx than in EXTREME, accounting for 11 (22%) versus 36 (67%), respectively (*p* < 0.001). Overall, 24 (44%) patients in the EXTREME and 6 (11%) patients in the UPEx arm developed ≥ grade 3 neutropenia (*p* < 0.001), while 17 (31%) patients in the EXTREME and 5 (9%) patients in the UPEx arm developed ≥ grade 3 anemia (*p* = 0.014). In the EXTREME arm, more than 40% patients had vomiting and oral mucositis, while in the UPEx arm, only 6% patients had vomiting and oral mucositis (*p* < 0.001). Meanwhile, 16 (30%) patients in the EXTREME arm and 3 (6%) in the UPEx arm developed diarrhea (*p* = 0.011). Approximately 20% patients in both arms developed a skin rash without significant difference (*p* = 0.517).

## 4. Discussion

To our best knowledge, this is the first study focusing on the oncologic outcomes of cetuximab-containing chemotherapy in elderly patients with R/M HNSCC. The preceding EXTREME study [6] only enrolled 18% of patients older than 65 years, and the TPEx study with taxane and platinum plus etuximab [8] excluded patients after the age of 70 years. More recently, KEYNOTE-048 found that pembrolizumab alone or in combination with platinum and 5-FU is an effective first-line treatment for programmed death—ligand 1-positive R/M HNSCC [9]. This famous study recruited few patients aged over 70 years. Therefore, little is known about whether these results can be used or not in elderly patients with R/M HNSCC [10]. Noor et al. summarized that aging and frailty have been demonstrated to be closely related to adverse outcomes, health-related quality of life, and treatment toxicity in R/M HNSCC patients [11]. Our study showed that UPEx is an effective regimen with tolerable toxic profiles for elderly patients. The median PFS and OS in the UPEx arm were 5.4 months and 10.8 months, while ORR and DCR were 61% and 72%, respectively, which were comparable with those of the EXTREME arm. Our conclusion can be a clinical reference for physicians who treat elderly patients with R/M HNSCC. Further prospective randomized controlled trials are warranted to validate our conclusions.

The elderly patient is a very heterogeneous patient type in relation to their general health state, degree of dependence, comorbidities, performance status, physical reserve, and geriatric situation [12]. Elderly patients with R/M HNSCC are at increased risk of adverse outcomes during and after treatment of head and neck cancer [4]. So, treatment in the elderly patient remains a therapeutic challenge. A comprehensive meta-analysis recruiting 93 head and neck cancer studies showed that age was a significantly predictive factor for severe late treatment related AEs [13]. These retrospective literatures consistently concluded that the survival outcomes were limited in elderly patients despite similar response rate to chemotherapy [14]. The possible explanations were increased treatment toxicity and non-cancer-related deaths in elderly patient with R/M HNSCC. Our study was also consistent with previous literature. Age and ECOG PS were strong prognostic factors related with survival. We also constructed a risk model by counting these two risk factors and divided patients into low-risk group and high-risk group. The oncologic outcomes were significantly different between these two groups. Our study constructed a reliable prognostic model, which is useful for outcome prediction, patient counseling, and risk stratification in clinical trials. Further prospective studies are warranted to validate our results.

The EXTREME regimen is one of the standard treatments for patients with R/M HNSCC, but the toxicity of the EXTREME regimen has limited its used in elderly patients. Within the EXTREME regimen, 5-FU is associated with more severe mucositis and diarrhea, and should not be used in patients with cardiovascular diseases or dihydropyrimidine dehydrogenase deficiency [15]. Furthermore, 5-FU is difficult to be administered due to 24 h continuous infusion for 4 days. The GORTEC group demonstrated OS was not significantly different between TPEx and EXTREME in patients with R/M HNSCC [16]. However, toxicity was significantly lower in TPEx, accounting for 34% patients who had grade ≥ 4 AEs versus 50% in EXTREME [8]. Fuchs et al. reported that modified biweekly TPEx was also an effective regimen and had lower rates of severe neutropenia, as compared to the conventional TPEx study [17]. Another phase II trial comparing paclitaxel in combination with carboplatin and cetuximab (PCE) with EXTREME concluded that PCE had similar efficacy and less toxicity compared to EXTREME (60% in EXTREME versus 40% in PCE; *p* = 0.034) [18,19]. More recently, immune checkpoint inhibitors have emerging roles and are changing the treatment landscape for R/M HNSCC patients. KEYNOTE-048 study demonstrated a similar toxicity profile to EXTREME when pembrolizumab is combined with chemotherapy, accounting for 85% grade 3 or worse all-cause AEs [20]. In our study, grade 3/4 AEs are much less in UPEx than in EXTREME, accounting for 22% vs. 67%, respectively (*p* < 0.001). Given a better safety profile with comparable efficacy, the UPEx regimen can be an alternative option for frail patients with R/M HNSCC who are intolerant of the EXTREME treatment.

There are several potential limitations in our work, which are inherent to any retrospective studies. Each chemotherapy regimen was selected by the preference of physicians. This is a major bias in this study. Meanwhile, a single institutional experience, a small sample size, heterogeneity of our patients, and inconsistent follow-up interval may also limit the power of our study. Our study investigated the oncologic outcomes of cetuximab-containing chemotherapy in elderly patients with R/M HNSCC. To date, there are no prospective randomized controlled trials with larger cohort focusing on the treatment of this group. In spite of a retrospective study with inevitable selection bias, our study remains clinically valuable.

## 5. Conclusions

Our study investigated the oncologic outcomes of cetuximab-containing chemotherapy in elderly patients with R/M HNSCC. Based on our results, we disclosed that both UPEx and EXTREME are both effective in elderly patients with R/M HNSCC. Furthermore, safety profiles are improved when UFUR is substituted for 5-FU. In our multivariate analysis, age and ECOG PS were strong prognostic factors related to survival. These conclusions are clinically valuable and pave the way for the treatment of elderly patients with R/M HNSCC. Further prospective randomized controlled trials are warranted to validate our conclusions.

## Figures and Tables

**Table 1:** Baseline characteristics of 152 patients with R/M HNSCC before and after PSM, stratified and chemotherapy regimens.

	Before PSM			After PSM		
	UPEx	EXTREME	*p*	UPEx	EXTREME	*p*
	N = 54	N = 98		N = 54	N = 54	
Gender			0.822			1
Male	51 (94%)	90 (92%)		51 (94%)	51(94%)	
Female	3 (6%)	8 (8%)		3 (6%)	3 (6%)	
Age			0.012			0.787
≤72	25 (65%)	82 (84%)		35 (65%)	34(63%)	
>72	19 (35%)	16 (16%)		19 (35%)	20(37%)	
ECOG PS			0.026			1
0–1	22 (41%)	58 (59%)		22 (41%)	22(41%)	
2	32 (59%)	40 (41%)		32 (59%)	32(59%)	
Smoking			0.493			0.512
Current/Former	51 (94%)	86 (88%)		51 (94%)	48(89%)	
Never	3 (6%)	12 (12%)		3 (6%)	6(11%)	
Primary tumor location			0.258			0.574
Oral cavity	20 (37%)	36 (37%)		20 (37%)	20 (37%)	
Oropharynx	8 (15%)	22 (22%)		8 (15%)	10 (19%)	
Hypopharynx	24 (44%)	36 (37%)		24 (44%)	22 (40%)	
Larynx	2 (4%)	4 (4%)		2 (4%)	2 (4%)	
Previous treatment history						
Radical surgery	37 (69%)	52 (81%)	0.306	37 (69%)	34 (63%)	0.442
Chemoradiotherapy	45 (83%)	88 (90%)	0.416	45 (83%)	44 (81%)	0.763
Disease status			0.331			0.483
Local recurrence only	15 (28%)	16 (16%)		15 (28%)	12 (22%)	
Distant metastasis	39 (72%)	82 (84%)		39 (72%)	42 (78%)	

R/M HNSCC, recurrent or metastatic head and neck squamous cell carcinoma; PSM, propensity score matching; UPEx, tegafur–uracil /cisplatin/cetuximab; EXTREME, 5-fluorouracil/cisplatin/cetuximab; ECOG PS, Eastern Cooperative Oncology Group Performance Status.

**Table 2:** Oncologic outcomes of 108 elderly patients with R/M HNSCC, stratified by chemotherapy regimen.

	UPEx N = 54	EXTREME N = 54	*p*
mPFS (m)	5.4	5.8	0.451
mOS (m)	10.8	10.2	0.807
CR (%)	0 (0)	0 (0)	
PR (%)	33 (61)	32 (59)	
SD (%)	6 (11)	6 (11)	
PD (%)	15 (28)	16 (30)	
ORR (%)	33 (61)	32 (59)	0.680
DCR (%)	39 (72)	38 (70)	0.732

R/M HNSCC, recurrent metastatic head and neck squamous cell carcinoma; UPEx, tegafur–uracil/cisplatin/cetuximab, EXTREME 5-fluorouracil/cisplatin/cetuximab; mPFS, median progression-free survival; mOS, median overall survival; CR, complete response; PR, partial response; SD, stable disease; PD, progressive disease; ORR, objective response rate; DCR, disease control rate.

**Table 3:** Cox regression analysis of parameters associated with survival.

	PFS		OS	
Variables	HR (95% CI)	*p* Value	HR (95% CI)	*p* Value
Age, ≤72 vs. >72	0.55 (0.26–0.89)	0.026	0.48 (0.19–0.76)	0.019
Sex, Male vs. Female	0.66 (0.15–2.17)	0.473	0.51 (0.12–1.78)	0.366
ECOG PS, 0–1 vs. 2	0.63 (0.36–0.85)	0.020	0.54 (0.23–0.84)	0.015
Smoking, no vs. yes	0.65 (0.27–1.34)	0.199	0.79 (0.31–1.63)	0.455
Primary location, oral cavity vs. others	0.96 (0.66–1.69)	0.746	0.74 (0.37–1.67)	0.598
Previous radical surgery, yes vs. no	0.89 (0.42–1.54)	0.491	0.71 (0.49–1.72)	0.513
Previous chemoradiotherapy, yes vs. no	0.71 (0.40–1.44)	0.393	0.79 (0.30–1.45)	0.342
Disease status, local only vs. metastasis	0.62 (0.31–1.34)	0.214	0.82 (0.35–1.88)	0.721
Chemotherapy, UPEx vs. EXTREME	0.75 (0.45–1.27)	0.286	0.89 (0.48–1.64)	0.711

PFS, progression-free survival; OS, overall survival; HR, hazard ratio; CI, confidence interval; ECOG PS, Eastern Cooperative Oncology Group Performance Status; UPEx, tegafur–uracil/cisplatin/cetuximab, EXTREME, 5-fluorouracil/cisplatin/cetuximab.

**Table 4:** Grade 3 to 4 treatment related adverse effects in 108 elderly patients with R/M HNSCC, stratified by chemotherapy regimen.

	UPEx N = 54	EXTREME N = 54	*p*
Hematologic events, n (%)			
Neutropenia	6 (11)	24 (44)	<0.001
Febrile neutropenia	2 (4)	10 (19)	0.039
Anemia	5 (9)	17 (31)	0.014
Non-hematologic events, n (%)			
Skin rash	10 (19)	12 (22)	0.517
Hypersensitivity	2 (4)	2 (4)	0.852
Diarrhea	3 (6)	16 (30)	0.011
Vomiting	3 (6)	24 (44)	<0.001
Oral mucositis	3 (6)	22 (41)	<0.001
Hand foot syndrome	5 (9)	6 (11)	0.737
Peripheral neuropathy	2 (4)	12 (22)	0.017

R/M HNSCC, recurrent metastatic head and neck squamous cell carcinoma; UPEx, tegafur-uracil/cisplatin/cetuximab; EXTREME, 5-fluorouracil/cisplatin/cetuximab.

## Data Availability

The datasets presented in this article are not readily available due to patient confidentiality and participant privacy terms. Requests to access the datasets should be directed to the corresponding author.
